# Use of Recombinant Zoster Vaccine in Immunocompromised Adults Aged ≥19 Years: Recommendations of the Advisory Committee on Immunization Practices — United States, 2022

**DOI:** 10.15585/mmwr.mm7103a2

**Published:** 2022-01-21

**Authors:** Tara C. Anderson, Nina B. Masters, Angela Guo, Leah Shepersky, Andrew J. Leidner, Grace M. Lee, Camille N. Kotton, Kathleen L. Dooling

**Affiliations:** ^1^Division of Viral Diseases, National Center for Immunization and Respiratory Diseases, CDC; ^2^Epidemic Intelligence Service, CDC; ^3^Immunization Services Division, National Center for Immunization and Respiratory Diseases, CDC; ^4^Stanford University School of Medicine, Stanford, California; ^5^Harvard Medical School, Boston, Massachusetts.

Zoster Vaccine Recombinant, Adjuvanted (Shingrix, GlaxoSmithKline [GSK]) is a 2-dose (0.5 mL each) subunit vaccine containing recombinant glycoprotein E in combination with adjuvant (AS01_B_) that was licensed in the United States for prevention of herpes zoster for adults aged ≥50 years by the Food and Drug Administration (FDA) and recommended for immunocompetent adults aged ≥50 years by the Advisory Committee on Immunization Practices (ACIP) in 2017* (*1*). On July 23, 2021, the FDA expanded the indication for recombinant zoster vaccine (RZV) to include adults aged ≥18 years who are or will be at increased risk for herpes zoster because of immunodeficiency or immunosuppression caused by known disease or therapy (*2*). On October 20, 2021, ACIP recommended 2 doses of RZV for the prevention of herpes zoster and related complications in adults aged ≥19 years^†^ who are or will be immunodeficient or immunosuppressed because of disease or therapy. RZV is the first herpes zoster vaccine approved for use in immunocompromised persons. With moderate to high vaccine efficacy and an acceptable safety profile, RZV has the potential to prevent considerable herpes zoster incidence and related complications. This report updates previous ACIP recommendations for the prevention of herpes zoster (*1*,*3*).

Herpes zoster is a painful, cutaneous eruption, usually involving one to three adjacent dermatomes,^§^ resulting from reactivation of latent varicella-zoster virus. The incidence of herpes zoster and related complications (including the most common complication of postherpetic neuralgia) increase with age (*3*–*5*). The risk for herpes zoster and related complications is generally higher in immunocompromised compared with immunocompetent adults, although there is heterogeneity within and across immunocompromised groups (*6*,*7*). The risk for herpes zoster among younger adults with certain immunocompromising conditions can be comparable to or higher than that in the general adult population aged >50 years (*6*,*7*). Because immunosuppression and immunodeficiency were contraindications for the previously available vaccine, zoster vaccine live,^¶^ and RZV was originally recommended for immunocompetent adults aged ≥50 years, there has been an unmet need for vaccination against herpes zoster in immunocompromised adults.

During December 2017–October 2021, the ACIP Herpes Zoster Work Group participated in monthly or bimonthly teleconferences to review herpes zoster epidemiology and evidence for the efficacy and safety of RZV in immunocompromised adults. These topics were discussed during four ACIP meetings in 2021. To guide its deliberations, ACIP used the Evidence to Recommendations Framework and the Grading of Recommendations, Assessment, Development and Evaluation (GRADE) approach (*8*) to evaluate possible benefits (prevention of herpes zoster, postherpetic neuralgia, and herpes zoster-related hospitalizations) and harms (serious adverse events [SAEs],** immune-mediated disease, graft-versus-host-disease, graft rejection, and reactogenicity) associated with RZV.^††^

Prevention of herpes zoster and occurrence of SAEs were deemed critical outcomes by the work group. Five studies in four immunocompromised groups^§§^ evaluated herpes zoster as an outcome (*9*–*13*). Estimates of vaccine efficacy (VE) came from three studies, with VE of 68.2% (95% CI = 55.6%–77.5%) for autologous hematopoietic cell transplant recipients (*11*), and 87.2% (44.3%–98.6%) and 90.5% (73.5%–97.5%) in post hoc efficacy analyses for patients with hematologic malignancies (*12*) and potential immune-mediated diseases (*13*), respectively. SAEs were evaluated in seven studies (*9*–*15*) in six immunocompromised groups (2,541 RZV recipients).^¶¶^ Overall, rates of SAEs were comparable between RZV and placebo recipients (risk ratios ranged from 0.79 to 1.99). SAEs deemed to be related to vaccination by study investigators ranged from 0% to 1.6% in the RZV group and 0% to 0.76% in the placebo group. The level of certainty for prevention of herpes zoster and occurrence of SAEs was type 2 (moderate).***

In addition to the critical outcomes (prevention of herpes zoster and SAEs), the remaining outcomes were deemed important by the work group. One study among hematopoietic cell transplant recipients (*11*) reported VE of 89% (95% CI = 22%–100%) for prevention of postherpetic neuralgia and 85% (32%–97%) for prevention of herpes zoster-related hospitalization (certainty type 3 [low]). Immune-mediated diseases were evaluated in six studies (*9*,*11*–*15*) in five immunocompromised groups^†††^ and were not increased among RZV recipients (certainty type 4 [very low]). One study in patients with hematologic malignancies (*12*) reported on graft-versus-host-disease among hematopoietic cell transplant recipients and did not identify an increased risk among RZV recipients (certainty type 4 [very low]). One study among renal transplant patients (*15*) reported on graft rejection and did not identify an increased risk among RZV recipients (certainty type 3). Local and systemic grade 3 reactions^§§§^ were evaluated in six studies (*9*–*12*,*14*,*15*) in five immunocompromised groups.^¶¶¶^ Local grade 3 reactions occurred in 10.7% to 14.2% of RZV recipients, and systemic grade 3 reactions occurred in 9.9% to 22.3% of RZV recipients, compared with 0% to 0.3% and 6.0% to 15.5%, respectively, among placebo recipients (certainty type 2).

Additional data reviewed within the Evidence to Recommendations Framework supported the use of RZV in immunocompromised adults.**** Two economic studies assessed RZV use (versus no vaccination) among immunocompromised adults (*16*). Both studies focused on hematopoietic cell transplant patients as the base case and found that vaccination was cost-saving, and eight to 10 persons receiving complete vaccination were needed to avert an episode of herpes zoster. Additional analyses assessed vaccination among persons with other immunocompromising conditions^††††^ and found that vaccination would cost <$99,000 per quality-adjusted life-year gained for most scenarios and could be cost-saving in several scenarios. Vaccination among patients with autoimmune and inflammatory conditions yielded the highest estimate of $208,000 per quality-adjusted life-year gained. Variations in results across scenarios were likely due to differences in estimated costs of health care, VE, and incidence of herpes zoster across different immunocompromising conditions.

Overall, the work group determined that herpes zoster in immunocompromised adults is of public health importance; the desirable anticipated effects of RZV in immunocompromised adults are large and the undesirable effects are small, which favors the intervention; immunocompromised adults probably feel that the desirable effects of vaccination with RZV are large relative to the undesirable effects and that there is probably not important uncertainty or variability in how patients value these outcomes. Use of RZV in immunocompromised adults is acceptable to stakeholders and a reasonable and efficient allocation of resources; health equity would probably be increased; and the intervention would be feasible to implement. On October 20, 2021, with this input from the work group, ACIP unanimously approved the recommendation.

With moderate to high VE among several immunocompromised groups and an acceptable safety profile across a range of immunocompromised groups, RZV has the potential to prevent considerable herpes zoster incidence and related complications. Recommending vaccination of immunocompromised adults aged ≥19 years will enable providers to vaccinate patients at a time most appropriate for their immunocompromising condition or therapy.

## Clinical Guidance^§§§§^

**Dosing schedule.** Two RZV doses are necessary, regardless of previous history of herpes zoster or previous receipt of zoster vaccine live. The second RZV dose should typically be given 2–6 months after the first; for persons who are or will be immunodeficient or immunosuppressed and who would benefit from a shorter vaccination schedule, the second dose can be administered 1–2 months after the first (*2*). If the second RZV dose is given sooner than 4 weeks after the first, a valid second dose should be repeated at least 4 weeks after the dose given too early. The vaccine series does not need to be restarted if more than 6 months have elapsed since the first dose.

**Timing of vaccination.** When possible, patients should be vaccinated before becoming immunosuppressed. Otherwise, providers should consider timing vaccination when the immune response is likely to be most robust (i.e., during periods of lower immunosuppression and stable disease). RZV may be administered to patients who previously received varicella vaccine. RZV is not a live virus vaccine; therefore, RZV may be administered while patients are taking antiviral medications.

**Coadministration with other vaccines.** Recombinant and adjuvanted vaccines, such as RZV, can be administered concomitantly, at different anatomic sites, with other adult vaccines, including COVID-19 vaccines (*17*). Concomitant administration of RZV with other adult vaccines^¶¶¶¶^ has been studied, and there was no evidence for interference in the immune response to either vaccine or of safety concerns (*18*–*20*). Coadministration of RZV with adjuvanted influenza vaccine (Fluad) and COVID-19 vaccines is being studied.

**Counseling for reactogenicity.** Before vaccination, providers should counsel patients about expected local and systemic reactogenicity, including grade 3 reactions. It is generally not recommended to take antipyretic or analgesic medications prophylactically before vaccination; however, antipyretic or analgesic medications may be taken for the treatment of postvaccination local or systemic symptoms. Patients should be encouraged to complete the series even if they experienced a (nonanaphylactic) grade 1–3 reaction after receipt of the first RZV dose.

## Special Populations^*****^

**Persons with a history of herpes zoster.** Herpes zoster can recur. Persons with a history of herpes zoster should receive RZV.

**Persons with no documented history of varicella, varicella vaccination, or herpes zoster.** Persons who have neither experienced varicella nor received varicella vaccine are not at risk for herpes zoster. More than 99% of Americans born before 1980 have had varicella (*21*). Children and adolescents who have received live-attenuated varicella vaccines are at lower risk for herpes zoster than are those who experienced varicella (*22,23*). RZV is not indicated and has not been studied for the prevention of varicella. For immunocompromised persons, evidence of immunity to varicella (confirming need for RZV) includes documented receipt of 2 doses of varicella vaccine, laboratory evidence of immunity or laboratory confirmation of disease, or diagnosis or verification of a history of varicella or herpes zoster by a health care provider. For immunocompromised adults with no documented history of varicella, varicella vaccination, or herpes zoster, providers should refer to the ACIP varicella vaccine recommendations for further guidance, including postexposure prophylaxis guidance (*24*).

**Pregnancy.** There is currently no ACIP recommendation for RZV use in pregnancy; therefore, providers should consider delaying RZV until after pregnancy. There is no recommendation for pregnancy testing before vaccination.

**Breastfeeding.** Recombinant vaccines such as RZV pose no known risk to mothers who are breastfeeding or to their infants (*17*). Clinicians may consider vaccination without regard to breastfeeding status if RZV is otherwise indicated.

## Contraindications

**Allergy.** RZV should not be administered to persons with a history of severe allergic reaction, such as anaphylaxis, to any component of this vaccine.

## Precautions

**Moderate or severe acute illness with or without fever.** In general, vaccination should be delayed for patients experiencing moderate or severe acute illness (*17*).

**Current episode of herpes zoster.** RZV is not a treatment for herpes zoster or postherpetic neuralgia. If a person is experiencing an episode of herpes zoster, vaccination should be delayed until the acute stage of the illness is over and symptoms abate (*17*).

## Reporting of Vaccine Adverse Events

Adverse events following vaccination can be reported to the Vaccine Adverse Events Reporting System (VAERS). Reporting is encouraged for any clinically significant adverse event even if it is uncertain whether the vaccine caused the event. Information on how to submit a report to VAERS is available at https://vaers.hhs.gov/index.html or by telephone at 1-800-822-7967.

## Future Research and Monitoring Priorities

CDC will monitor adverse events following RZV immunization through VAERS, the Vaccine Safety Datalink, and observational studies. This is particularly important given the heterogeneity of herpes zoster risk within and across immunocompromised groups and the novel adjuvant and high rates of reactogenicity of the vaccine. Limited data for outcomes deemed important by the work group (e.g., possible graft rejection, graft-versus-host-disease, immune-mediated disease) highlight the need for additional research. Additional post-marketing monitoring will include studies conducted by GSK and reported to FDA. Continued monitoring of the impact of the U.S. varicella and herpes zoster vaccination programs on herpes zoster epidemiology will be important to guide future herpes zoster vaccination recommendations.

**Summary**

**What is already known about this topic?**

Immunocompromised persons experience a higher incidence of herpes zoster and related complications. On July 23, 2021, the Food and Drug Administration expanded the indication for use of recombinant zoster vaccine (RZV) to include immunodeficient or immunosuppressed adults.

**What is added by this report?**

On October 20, 2021, the Advisory Committee on Immunization Practices recommended 2 RZV doses for prevention of herpes zoster and related complications in immunodeficient or immunosuppressed adults aged ≥19 years.

**What are the implications for public health practice?**

RZV is the first herpes zoster vaccine approved for use in immunocompromised persons. With moderate to high vaccine efficacy and an acceptable safety profile, RZV has the potential to prevent considerable herpes zoster incidence and related complications.

## Footnotes

* This recommendation became official CDC policy in January 2018.
